# Immune-mediated renal injury in diabetic kidney disease: from mechanisms to therapy

**DOI:** 10.3389/fimmu.2025.1587806

**Published:** 2025-06-04

**Authors:** Lingli Ma, Dianyuan Liu, Yue Yu, Zimeng Li, Qing Wang

**Affiliations:** Department of Endocrinology and Metabolism, China-Japan Union Hospital of Jilin University, Changchun, China

**Keywords:** immune cell, diabetic kidney disease, mechanisms, treatment methods, immunotherapy

## Abstract

Diabetic kidney disease (DKD) is now recognized as a multifactorial disorder, driven by the interplay of metabolic dysfunction, chronic inflammation, and immune-mediated renal injury. This review comprehensively synthesizes recent advancements in understanding immune dysregulation as a central driver of DKD pathogenesis, integrating molecular mechanisms with emerging therapeutic strategies. Innate immune activation, which includes macrophage polarization and adaptive immune perturbations, exacerbates glomerulosclerosis and interstitial fibrosis through cytokine storms and mitochondrial oxidative stress. Despite clinical guidelines emphasizing glycemic control and renin-angiotensin-aldosterone system (RAAS) inhibition, their limited efficacy in halting immune-mediated tubular atrophy highlights the unmet need for targeted immunotherapies. By connecting mechanistic discoveries to clinical translation, this work establishes a roadmap for the development of immune-centric therapies. Its critical synthesis of multi-omics data, clinical trial evidence, and preclinical models bridges the gap between laboratory discoveries and bedside applications, laying the groundwork for redefining DKD as a treatable immune-metabolic disorder.

## 1 Introduction

DKD, characterized by persistent albuminuria and a progressive decline in the glomerular filtration rate among diabetic patients (1), Epidemiological studies estimate that over 40% of diabetic patients develop renal complications within 15 years of diagnosis, significantly contributing to cardiovascular morbidity and mortality (2), remaining the predominant cause of chronic kidney disease and end-stage renal failure (3). The pathogenesis of DKD arises from multifaceted interactions among metabolic disturbances, inflammatory cascades, and fibrotic remodeling mediated by Transforming Growth Factor Beta (TGF-β) signaling (4, 5). Historically, hemodynamic factors and podocyte injury have been emphasized, but emerging evidence highlights the role of both innate and adaptive immune responses in disease progression (6). Notably, activated macrophages infiltrate the kidneys of diabetic patients, triggering cytokine storms that perpetuate tubular injury and interstitial fibrosis (7, 8). These discoveries redefine DKD not only as an immunometabolic disorder but also highlight potential drug targets to halt the progression of the disease.

The hyperglycemic environment in diabetes triggers glomerular hyperfiltration and tubular epithelial damage (9), triggering renal RAAS activation exacerbates monocyte/macrophage infiltration. This pathogenic cascade amplifies pro-inflammatory cytokine networks, driving extracellular matrix deposition through TGF-β1/Smad3 signaling, thereby accelerating glomerulosclerosis and tubulointerstitial fibrosis (10). Clinical biopsies reveal that elevated levels of tumor necrosis factor-alpha (TNF-α) correlate with the severity of albuminuria in diabetic patients (11), while IL-6 activates signal transducer and activator of transcription 3 (STAT3), thereby promoting the proliferation of renal fibroblasts (12). Immune cells play a dual role in the progression of DKD: they are involved in the protective clearance of apoptotic debris as well as the maladaptive perpetuation of chronic inflammation (13). CD4+ T-cells infiltrate diabetic kidneys through the activation of the chemokine ligand 20 (CCL20)/chemokine receptor 6 (CCR6) axis, secreting interferon gamma (IFN-γ) to recruit M1 macrophages that exacerbate oxidative stress (14, 15). Paradoxically, IL-10 derived from B-cells mitigates tubular injury while simultaneously increasing autoantibodies against basement membrane components (16). Regulatory T-cells (Tregs) are crucial in modulating this balance; their functional impairment disrupts IL-35-mediated immunosuppression, leading to Th17-driven interstitial inflammation (17). These findings emphasize the therapeutic potential of selectively targeting immune checkpoints while maintaining renal immunoregulatory functions.

Current therapeutic approaches for DKD primarily focus on the use of RAAS inhibitors and Sodium-Glucose Cotransporter 2 (SGLT-2) inhibitors, aimed at decelerating glomerular hyperfiltration and diminishing proteinuria (18). However, these treatments do not fully control disease progression, with over 30% of patients still experiencing progressive renal function decline due to unresolved tubulointerstitial inflammation and macrophage-driven fibrosis. This limitation arises from the current paradigm’s insufficient targeting of immune-inflammatory pathways — a mechanism increasingly recognized in the pathogenesis of DKD (6, 19, 20). Emerging evidence indicates that immune modulation is a strategic frontier: preclinical models demonstrate that NOD-like receptor protein 3 (NLRP3) inflammasome inhibitors decrease Interleukin-1β (IL-1β) levels and mitigate podocyte injury (21), Targeting monocyte chemoattractants with chemokine receptor 2 (CCR2) antagonists results in a significant reduction in urinary excretion of monocyte chemoattractant protein 1 (MCP-1) (22). Specifically, modulation of macrophage polarization through inhibition of the TGF-β/Smad3 pathway has been demonstrated to reduce collagen deposition in the kidneys of diabetic rodents (23). T-cell subset manipulation also shows promise; adoptive transfer of Tregs in DKD rat models restored the Th17/Treg balance and reduced albuminuria (24). These advances necessitate a paradigm shift—from viewing DKD solely as a metabolic disorder to embracing its immune-mediated pathophysiology in therapeutic innovation.

In summary, the pathogenesis of DKD is complex, with the immune system playing a crucial role in its development. Future research should further investigate the specific mechanisms by which immune cells contribute to DKD, as well as explore how modulating immune responses can improve patient prognosis. By gaining a deeper understanding of the pathogenesis of DKD, we aim to develop more effective therapeutic strategies, ultimately enhancing the quality of life and health outcomes for patients with diabetes.

## 2 Pathogenic mechanisms of DKD

### 2.1 Impact of hyperglycemia

**Figure 1:**
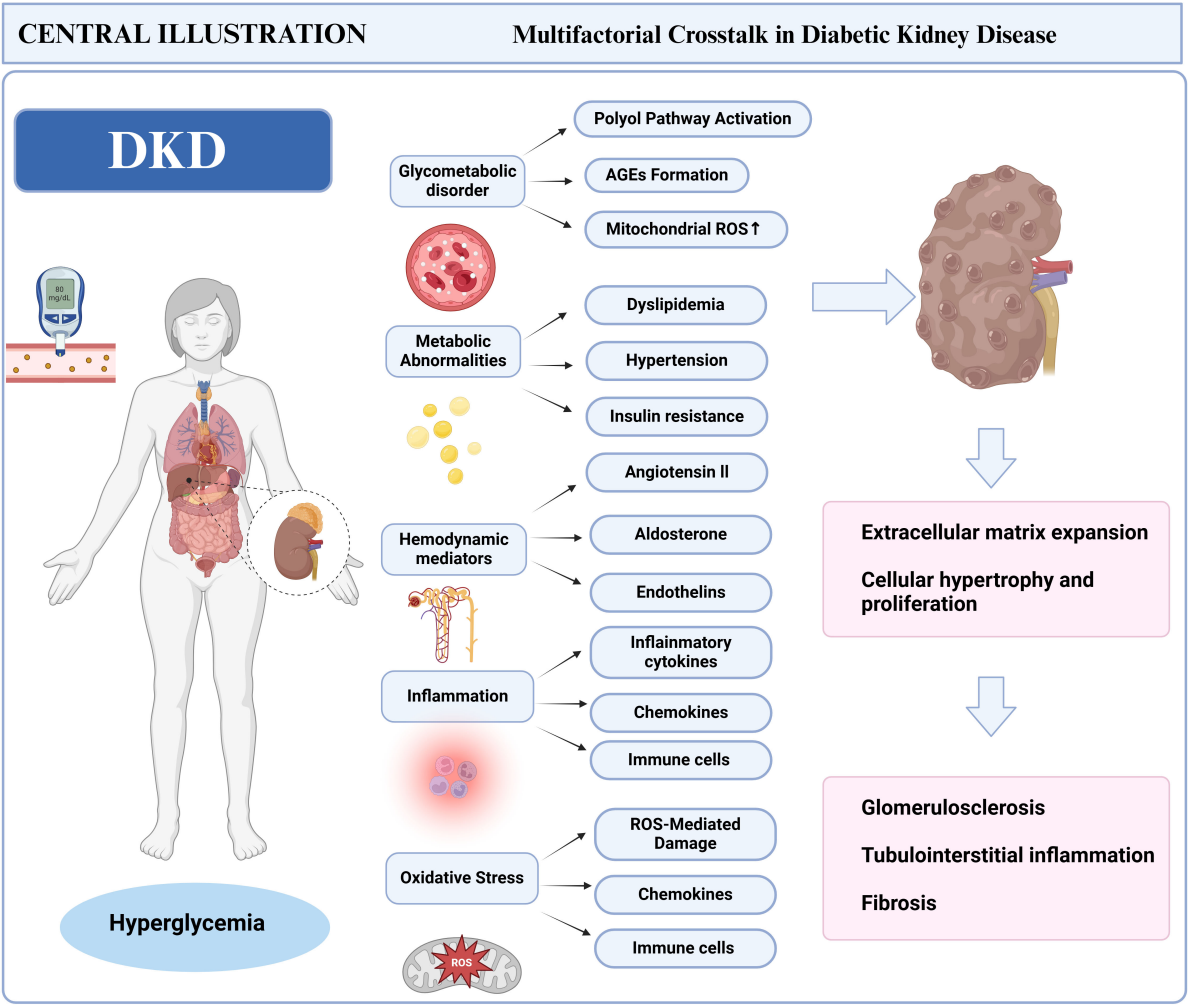
Pathogenic mechanisms of DKD.

Persistent hyperglycemia acts as a crucial catalyst for DKD, initiating pathological cascades via prolonged metabolic dysregulation and cellular injury (25, 26). Elevated glucose levels trigger the polyol pathway, in which aldose reductase facilitates the conversion of glucose into sorbitol and fructose (27). Sorbitol accumulation disrupts cellular osmotic equilibrium, inducing intracellular swelling and functional impairment in renal tubular and glomerular cells. Concurrently, excess glucose promotes the formation of advanced glycation end products (AGEs) (28), These substances bind to receptors on renal cells, activating nuclear factor-κB (NF-κB) and reactive oxygen species (ROS) signaling pathways. These molecular events promote chronic inflammation, endothelial dysfunction, and apoptosis—key features of DKD progression. Notably, AGE-induced oxidative stress exacerbates podocyte loss and thickening of the glomerular basement membrane (GBM), accelerating albuminuria and interstitial fibrosis. Emerging evidence highlights a feedback loop between hyperglycemic ROS overproduction and insulin resistance, perpetuating renal injury through mitochondrial dysfunction and reduced nitric oxide bioavailability (29, 30) (**Figure 1**).

Hyperglycemia further disrupts immune responses via multi-level metabolic disturbances: renal intrinsic cells, including tubular epithelial and mesangial cells, secrete MCP-1 and intercellular adhesion molecule-1 (ICAM-1) under sorbitol-induced osmotic stress and endoplasmic reticulum stress, specifically recruiting circulating monocytes to differentiate into pro-inflammatory M1 macrophages. The interaction between AGEs and their receptors not only activates NF-κB to drive an explosive secretion of cytokines but also forms a “cytokine storm” that chemotactically recruits CD4+ T cells into the renal interstitium, amplifying local inflammation. Under hyperglycemic conditions, elevated diacylglycerol (DAG) activates protein kinase C (PKC). This activation not only promotes mesangial cell proliferation and extracellular matrix deposition but also enhances NF-κB nuclear translocation through phosphorylation, thereby sustaining inflammatory signaling cascades (31). Mitochondrial dysfunction serves as a critical amplifier: excessive glucose oxidation leads to a burst production of mitochondrial ROS, which directly damages glomerular endothelial cells and podocytes while oxidatively modifying Toll-like receptor 4 (TLR4), enhancing macrophage sensitivity to pathogen-associated molecular patterns (PAMPs) and triggering sterile inflammatory responses (32). This metabolism-immunity crosstalk disrupts both innate and adaptive immunity: it drives the aberrant activation of macrophages and neutrophils while reprogramming T cell metabolism—enhancing glycolysis in pro-inflammatory Th1/Th17 cells and impairing mitochondrial oxidative phosphorylation in Tregs—ultimately establishing a chronic intra-renal inflammatory microenvironment that accelerates glomerulosclerosis and tubulointerstitial fibrosis.

Podocyte injury serves as a critical connection between hyperglycemia and immune activation in DKD. Oxidative stress and metabolic dysfunction, induced by hyperglycemia, directly lead to podocyte apoptosis, resulting in the release of podocyte-specific antigens and damage-associated molecular patterns (DAMPs), including high-mobility group box 1 (HMGB1) and ATP (33). The recognition of these molecules by renal macrophages triggers the TLR4/NF-κB pathway, leading to M1 polarization and the secretion of pro-inflammatory cytokines, which further intensifies tubulointerstitial inflammation. Clinical studies and preclinical models suggest that Tim-3-expressing macrophages, whose renal expression correlates with the severity of diabetic nephropathy, induce podocyte injury through the NF-κB/Tumor necrosis factor-alpha (TNF-α) pathway, with reduced nephrin expression being inversely associated with macrophage activation (34). Mechanistically, the phagocytosis of apoptotic podocyte debris by macrophages triggers the activation of the NLRP3 inflammasome, which in turn promotes the maturation of IL-1β and IL-18, thereby establishing a “podocyte-macrophage” interactive damage cycle (35).

### 2.2 Metabolic abnormalities and kidney injury

Metabolic disturbances, including dyslipidemia, hypertension, and insulin resistance, synergistically exacerbate renal injury in DKD. Hyperlipidemia promotes intracellular lipid accumulation in renal tubular cells, triggering lipotoxicity through mitochondrial dysfunction and endoplasmic reticulum stress (36, 37). Excessive free fatty acids (FFAs) activate peroxisome proliferator-activated receptors (PPARs) and sterol regulatory element-binding proteins (SREBPs), which drive pro-apoptotic pathways and fibrosis through TGF-β1/Smad3 signaling (38, 39). Simultaneously, the components of metabolic syndrome exacerbate oxidative stress by upregulating nicotinamide adenine dinucleotide phosphate (NADPH) oxidase (NOX) isoforms, further damaging glomerular endothelial cells and podocytes. Byproducts of lipid peroxidation, such as malondialdehyde (MDA) and 4-hydroxynonenal (4-HNE), directly modify renal cellular proteins, impairing autophagy and promoting tubular interstitial fibrosis (40). Importantly, hypertensive conditions in diabetes increase glomerular capillary pressure via angiotensin II-mediated vasoconstriction, accelerating albuminuria and mesangial matrix expansion. These metabolic cascades intersect with pathways induced by hyperglycemia, creating a vicious cycle that drives progressive nephron loss.

### 2.3 Elevated PKC activity in diabetic kidney disease

PKC is a critical mediator of renal injury in DKD (41). Under hyperglycemic conditions, elevated intracellular glucose levels lead to the synthesis of diacylglycerol (DAG), a potent activator of PKC isoforms, including PKC-α and PKC-β. The activation of PKC disrupts cellular signaling cascades, directly contributing to glomerular hypertrophy by promoting mesangial cell proliferation and extracellular matrix (ECM) deposition (42). For example, PKC-β enhances the expression of TGF-β1, which stimulates the synthesis of collagen IV and fibronectin, accelerating the thickening of the glomerular basement membrane and interstitial fibrosis (43). Additionally, PKC signaling exacerbates endothelial dysfunction by upregulating vascular endothelial growth factor (VEGF), leading to altered renal microvascular permeability and albuminuria (44). This pathway also contributes to oxidative stress by activating NADPH oxidase, which generates ROS that further damage podocytes and tubular cells. Notably, activation of PKC-δ phosphorylates pro-inflammatory transcription factors such as NF-κB, amplifying cytokine production and sustaining renal inflammation (45). These interconnected mechanisms highlight PKC as a crucial therapeutic target for reducing fibrosis and functional decline in diabetic kidney disease.

### 2.4 Oxidative stress imbalance in DKD

Oxidative stress is a key contributor to renal injury in DKD (46). Hyperglycemia stimulates the overproduction of ROS via mitochondrial dysfunction, NOX activation, and the AGE-mediated pathways (47). Excessive ROS cause oxidative damage to cellular components, such as lipid membranes, proteins, and DNA, thereby inducing podocyte apoptosis and tubular epithelial cell necrosis (48). Notably, overproduction of mitochondrial ROS in glomerular endothelial cells disrupts intercellular communication with podocytes, exacerbating albuminuria. Oxidative stress also activates pro-inflammatory NF-κB signaling, increasing renal expression of IL-6 and TNF-α, which perpetuate inflammation and fibrosis. Clinical studies reveal elevated urinary 8-hydroxy-2’-deoxyguanosine (8-OHdG), a biomarker of oxidative DNA damage (49), correlates with declining glomerular filtration rates in DKD patients. These data underscore oxidative stress as both a contributor to and a consequence of metabolic dysregulation in diabetic renal injury.

## 3 Role of immune cells in the pathogenesis of DKD

### 3.1 Macrophages

Macrophages, which originate from circulating monocytes, are central innate immune cells that polarize into pro-inflammatory M1 or anti-inflammatory/pro-fibrotic M2 phenotypes in response to microenvironmental cues (50). Under physiological conditions, renal-resident macrophages sustain tissue homeostasis by phagocytosing debris, regulating extracellular matrix turnover, and secreting reparative cytokines such as IL-10. In diabetic kidneys (51), hyperglycemia and AGEs prompt renal tubular cells to excessively express chemokines and adhesion molecules, thereby facilitating the recruitment of monocytes and the infiltration of macrophages into the glomeruli and interstitium.

In the early stages of DKD, M1 macrophages predominate, secreting pro-inflammatory cytokines and ROS. These substances exacerbate glomerular endothelial damage, podocyte apoptosis, and tubulointerstitial inflammation (52). These mediators perpetuate leukocyte recruitment and activate renal parenchymal cells to secrete additional inflammatory factors, creating a self-sustaining inflammatory loop. In contrast, late-stage DKD is characterized by the accumulation of M2 macrophages (53), which promotes fibrogenesis through TGF-β1-induced collagen synthesis and the activation of myofibroblasts, ultimately resulting in glomerulosclerosis and tubulointerstitial fibrosis. Notably, galectin-3 derived from M2 macrophages has been identified as a key regulator of renal stiffening in diabetic contexts.

**Figure 2:**
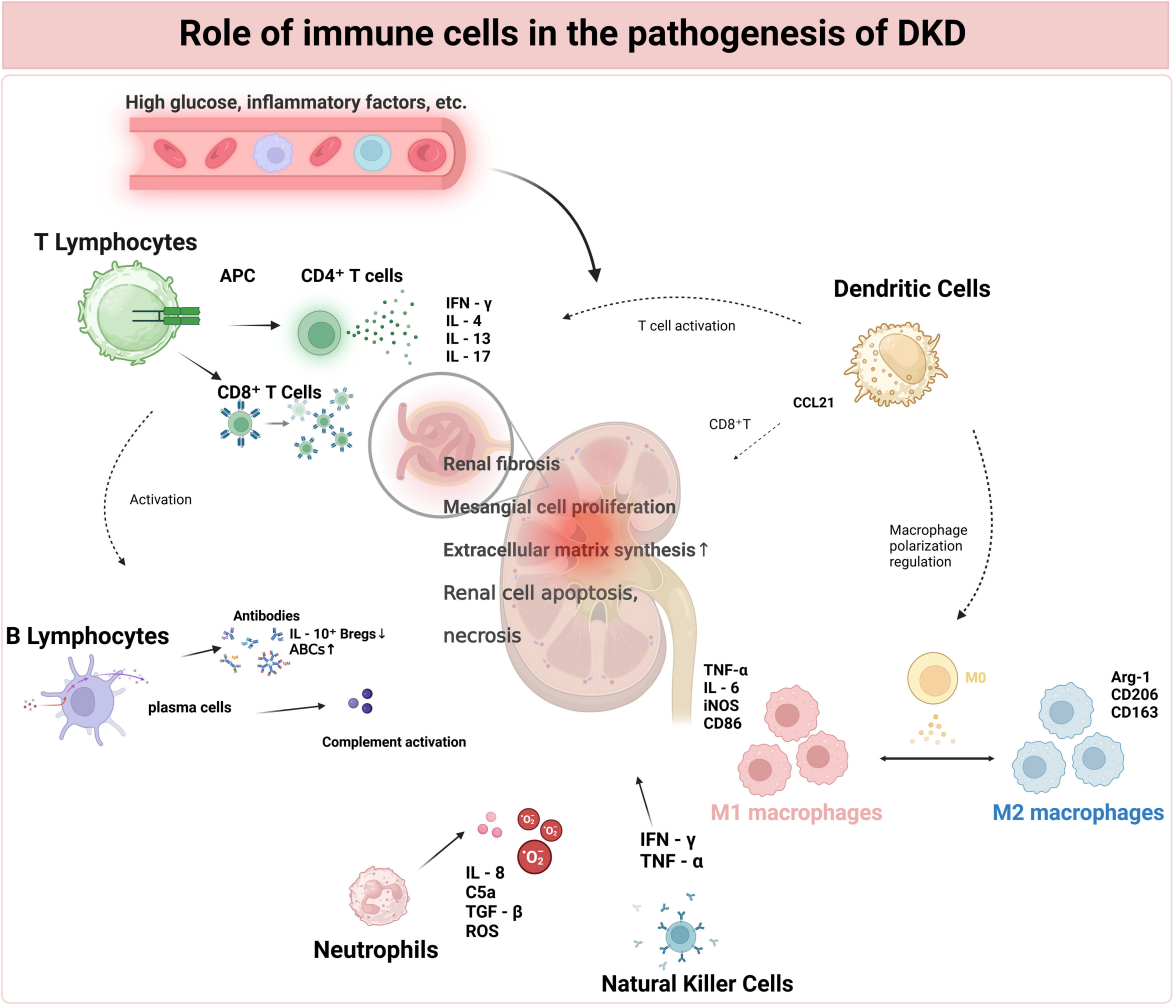
Role of immune cells in the pathogenesis of DKD.

Macrophage polarization dynamically interacts with the progression of DKD through bidirectional mechanisms. Hyperglycemia- and AGE-induced oxidative stress skew macrophages toward the M1 phenotype via NF-κB/STAT1 signaling (54), while chronic hypoxia in advanced disease enhances M2 polarization via hypoxia-inducible factor-1α (HIF-1α). Cytokines derived from M1 macrophages impair tubular autophagy, exacerbating inflammatory injury, whereas M2-mediated fibrosis disrupts vascular architecture, creating hypoxic niches that further drive M2 polarization (55, 56). Targeting macrophage plasticity through CCR2 inhibition or PPARγ agonists shows therapeutic potential by reducing both inflammation and fibrosis in preclinical models (57). This phenotypic plasticity underscores macrophages as the central orchestrators of immune-mediated renal injury in DKD (**Figure 2**).

Autophagic dysfunction is pivotal in the dysregulation of macrophages induced by hyperglycemia. High glucose suppresses autophagic flux in renal tubular epithelial cells through the mammalian target of rapamycin (mTOR)/Unc-51-like kinase 1 (ULK1) pathway, resulting in the accumulation of undegraded protein aggregates and damaged mitochondria (58). This triggers the release of DAMPs, which activate TLR/NF-κB signaling in macrophages to promote M1 polarization. Mechanistically, hyperglycemia enhances M1 macrophage polarization through the miR-32/Mef2d/cAMP pathway, directly upregulating pro-inflammatory genes while downregulating autophagy markers, creating a “autophagic defect-inflammatory amplification” loop (59). Additionally, impaired autophagy in Tregs disrupts mitochondrial bioenergetics, reducing FoxP3 expression and weakening suppression of Th17 cells, further exacerbating renal inflammation (60).

T lymphocytes, which include CD4^+^ T cells activated by a high-glucose environment and APCs, as well as CD8^+^ T cells activated by antigens and cytokines, play crucial roles. CD4^+^ T cells differentiate into Th1, Th2, and Th17 cells, which secrete cytokines such as IFN-γ, IL-4, and IL-17, respectively, each with distinct effects on inflammation and renal tissue. B lymphocytes, activated by antigens and T-cell assistance, form immune complexes that activate the complement system. Macrophages, recruited by high glucose and inflammatory factors, polarize into M1 and M2 types. DCs present antigens to T cells and recruit CD8^+^ T cells via CCL21. Neutrophils, recruited by inflammatory factors, damage renal tissue. NK cells, activated by cytokines, may have dysregulated functions in DKD. All these immune-cell-mediated effects converge, leading to glomerular and tubulo-interstitial lesions, ultimately resulting in proteinuria, a decline in renal function, and the development of DKD. Abbreviations: DKD (Diabetic Kidney Disease), APC (Antigen-Presenting Cells), IFN-γ (Interferon-γ), IL-4 (Interleukin-4), IL-13 (Interleukin-13), IL-17 (Interleukin-17), DC (Dendritic Cells), NK Cells (Natural Killer Cells).

### 3.2 T cells in DKD pathogenesis

T lymphocytes, which originate from bone marrow progenitors and differentiate in the thymus, consist of heterogeneous subsets such as CD4+ helper T cells (Th), cytotoxic CD8+ T cells (CTLs), and Tregs (61). These subsets regulate immune homeostasis by balancing pathogen clearance and self-tolerance (62). In DKD, adaptive immune dysregulation disrupts this balance: CD4+ T-cell infiltration in the glomeruli and tubulointerstitium correlates with the severity of albuminuria (7). Th1 cells drive pro-inflammatory responses through the secretion of IFN-γ and TNF-α, which promotes tubular apoptosis and podocyte loss. Meanwhile, Th17 cells secrete IL-17, recruiting neutrophils and exacerbating glomerular injury (63). Concurrently, hyperglycemia-induced oxidative stress impairs Treg function, reducing the production of TGF-β and IL-10, thereby weakening anti-inflammatory regulation (64). This imbalance between pro-inflammatory Th1/Th17 cells and suppressive Tregs creates a pathologic loop that sustains renal inflammation (65, 66).

The functional dichotomy of T-cell subsets is central to the progression of DKD. Th1 cells amplify macrophage activation through IFN-γ (67), enhancing M1 polarization and accelerating glomerulosclerosis. Th17-derived IL-17 stimulates fibroblasts to secrete collagen, directly promoting tubulointerstitial fibrosis. Cytotoxic CD8+ T cells induce tubular epithelial cell death via perforin and granzyme B, contributing to interstitial damage (68). Additionally, metabolic reprogramming in T cells under diabetic conditions—shifting from oxidative phosphorylation to glycolysis—elevates ROS production, further impairing Treg function and aggravating immune dysregulation (69). Clinical evidence indicates that increased Th17/Treg ratios and CD8+ cell infiltration are associated with a decrease in estimated glomerular filtration rate (eGFR) and proteinuria, underscoring their prognostic importance (70). Therapeutic approaches aimed at T-cell polarization, such as restoring Treg function or suppressing Th17 signaling, have shown promise in preclinical DKD models, suggesting potential pathways for immune modulation.

### 3.3 Dendritic cells in DKD pathogenesis

Dendritic cells (DCs), which originate from bone marrow precursors, are professional antigen-presenting cells that connect innate and adaptive immunity. They differentiate into subpopulations, including conventional DCs (cDCs) and plasmacytoid DCs (pDCs), each with unique tissue-specific niches in the kidney (71, 72). cDCs activate T cells through MHC-mediated antigen presentation, whereas pDCs secrete type I interferons during viral responses. Under hyperglycemic conditions, renal DCs mature through TLR4/NF-κB signaling, upregulating co-stimulatory molecules (CD80/86) and secreting pro-inflammatory cytokines, thereby amplifying local inflammation (73). Their dual role in immune surveillance and tolerance disruption establishes DCs as central mediators of DKD immunopathology.

Renal DCs drive glomerular injury by recruiting cytotoxic CD8+ T cells via CCL21 and promoting podocyte apoptosis through FasL-Fas interactions. In tubular interstitium, DC-derived TGF-β1 and IL-23 activate fibroblasts, accelerating collagen deposition and fibrosis (74). Additionally, perivascular DCs exacerbate endothelial dysfunction by upregulating adhesion molecules and facilitating monocyte infiltration. Single-cell RNA sequencing reveals CKD patients’ kidneys had decreased CD16+ NK cells while CD4+ naive helper T cells and CCR7+ DC increased, correlating with microvascular rarefaction and albuminuria progression (75). Therapeutic targeting of DC plasticity, such as blocking IL-6/STAT3 signaling, may restore immune balance and mitigate renal damage in DKD (76, 77).

### 3.4 B cells in DKD pathogenesis

B cells, which originate from hematopoietic stem cells in the bone marrow, are essential components of the adaptive immune system (78). They differentiate into plasma cells that secrete antibodies and act as antigen-presenting cells (APCs) through interactions mediated by major histocompatibility complex class II (MHC-II) with T cells (79). Subsets of B cells include regulatory B cells (Bregs), which produce anti-inflammatory cytokines, and follicular B cells, which drive germinal center responses. In the context of renal homeostasis, Bregs help to reduce tissue injury by suppressing autoimmune reactions via programmed death-ligand 1 (PD-L1) signaling and by enhancing immune complex clearance through Fc gamma receptor IIb (FcγRIIb) receptors (80). Furthermore, B cells contribute to maintaining local tolerance by promoting the differentiation of Tregs and by scavenging apoptotic debris in the glomeruli to prevent the formation of autoantibodies.

In DKD, chronic hyperglycemia induces B cell hyperactivity, characterized by elevated autoantibodies that cross-react with glomerular antigens, thereby triggering complement activation and podocyte injury. Non-antibody-dependent mechanisms involve aberrant B cell cytokine secretion, which exacerbates endothelial dysfunction by upregulating adhesion molecules and recruiting macrophages to the renal interstitium (81). Therapeutic strategies targeting B cell depletion or Breg augmentation demonstrate potential in reversing renal inflammation in preclinical models (82).

### 3.5 Other immune cells

Beyond T cells, B cells, macrophages, and DCs, emerging evidence implicates neutrophils, mast cells, and the complement system in the progression of DKD. Neutrophils infiltrate diabetic kidneys via ICAM-1/MCP-1-mediated chemotaxis, releasing neutrophil extracellular traps (NETs) enriched in histones and proteases that induce podocyte detachment and tubular injury (83, 84). exacerbate renal fibrosis by mediating the activation of TGF-β1 through tryptase and driving vascular permeability via histamine (85). The complement system is chronically activated in hyperglycemic microenvironments, where complement components 3a (C3a) and C5a anaphylatoxins enhance macrophage chemotaxis and stimulate pro-inflammatory cytokine production, amplifying glomerular endothelial damage (86, 87). Moreover, neuro-immune crosstalk that involves splenic sympathetic activation fosters systemic inflammation, which in turn drives renal immune cell infiltration. Focusing on these less-studied immune components could provide new therapeutic approaches.

## 4 Current and emerging therapeutic strategies

### 4.1 Current pharmacological treatment

#### 4.1.1 Insulin

**Figure 3:**
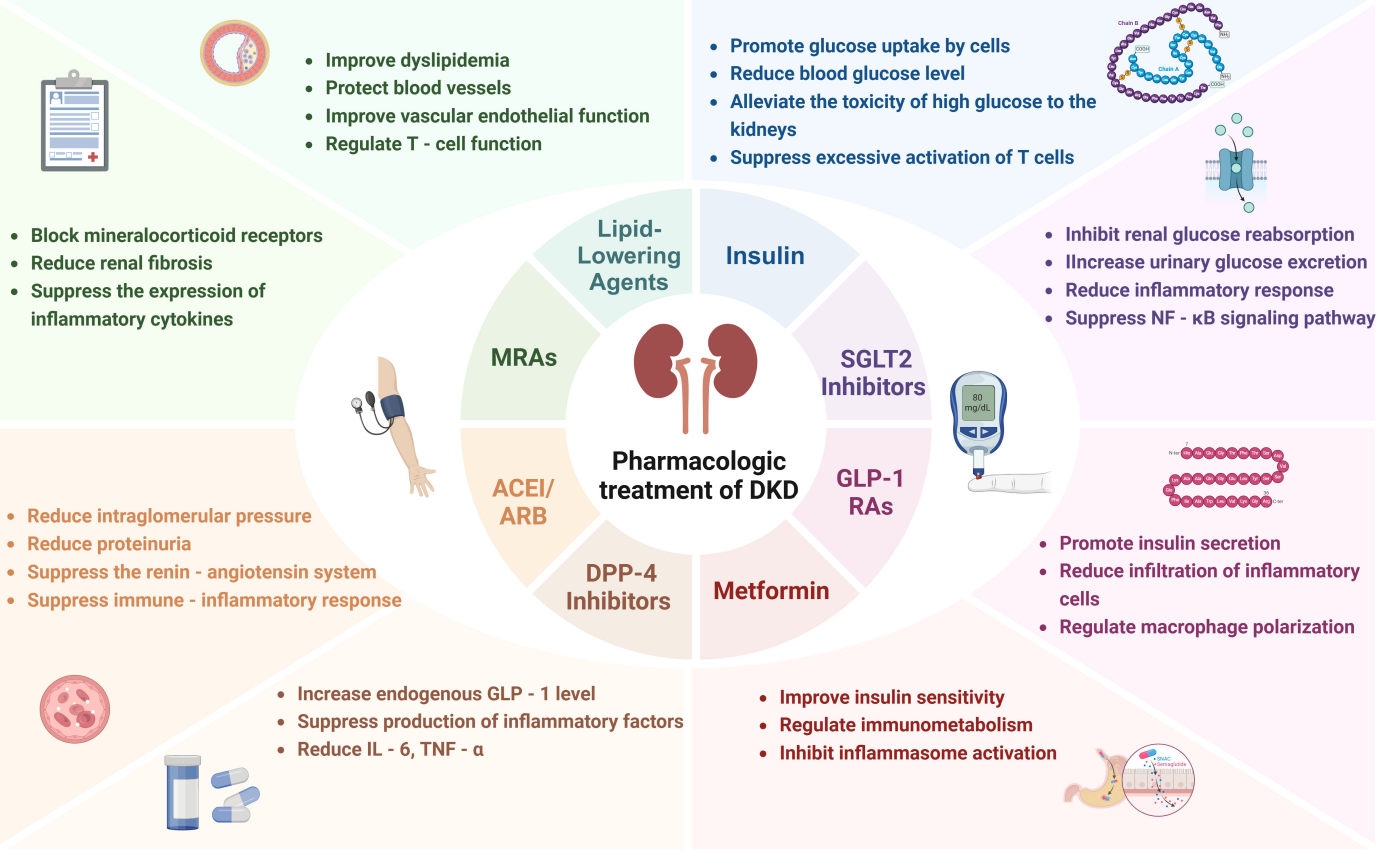
Current pharmacologic treatment of DKD.

Insulin therapy continues to be a fundamental element in the management of DKD, primarily due to its essential function in glycemic control. By achieving normoglycemia, insulin effectively decreases the accumulation of AGEs and mitigates mitochondrial oxidative stress, consequently alleviating glomerular hyperfiltration and preventing podocyte loss (88). The Diabetes Control and Complications Trial (DCCT) demonstrated that intensive insulin regimens reduce the progression of albuminuria and delay microvascular complications in type 1 diabetes (89). Emerging evidence underscores the immunomodulatory effects in the pathogenesis of DKD. Hyperglycemia-induced interactions between AGEs and their receptor, receptor for advanced glycation end products (RAGE), activate NF-κB signaling in renal macrophages, thereby amplifying pro-inflammatory cytokines and chemokines that recruit monocytes to the kidney. Insulin counteracts this by suppressing NLRP3 inflammasome activation in macrophages, which in turn reduces the secretion of IL-1β and IL-18, cytokines associated with tubular injury (90). Beyond glycemic control, insulin promotes podocyte autophagy through the phosphatidylinositol 3-kinase/protein kinase B (PI3K/AKT) pathway, thereby mitigating high glucose-induced apoptosis and safeguarding cytoskeletal proteins such as nephrin, which in turn diminishes macrophage recognition of damaged podocytes (91) (**Figure 3**).

#### 4.1.2 SGLT2 inhibitors

SGLT2 inhibitors, initially developed for glycemic control, exert renoprotection in DKD through traditional mechanisms involving hemodynamic modulation and metabolic improvements. By inhibiting glucose reabsorption in the proximal tubule, these agents reduce renal hyperfiltration—a hallmark of early DKD—via tubuloglomerular feedback, thereby lowering intraglomerular pressure and albuminuria. The Canagliflozin and Renal Endpoints in Diabetes with Established Nephropathy Clinical Evaluation (CREDENCE) trial demonstrated that canagliflozin reduced the risk of composite renal outcomes and lowered urinary albumin-to-creatinine ratio in patients with DKD (92). Additionally, SGLT2 inhibitors promote natriuresis and weight loss, indirectly mitigating obesity-related renal inflammation (93).

Beyond these classical effects, emerging evidence underscores their immunomodulatory actions. SGLT2 inhibitors mitigate renal NLRP3 inflammasome activation, which is hyperstimulated in diabetic kidneys, thereby decreasing IL-1β and IL-18 secretion from macrophages (94). They also suppress the AGE-RAGE-NF-κB axis, downregulating pro-inflammatory cytokines and chemokines, which are critical for monocyte recruitment and interstitial fibrosis. Preclinical studies indicate that these drugs shift macrophage polarization from a pro-inflammatory M1 to an anti-inflammatory M2 phenotype through HIF-1α downregulation and AMPK activation, enhancing tissue repair and reducing oxidative stress (95, 96). Clinically, dapagliflozin has been shown to reduce urinary MCP-1 levels in patients with DKD, correlating with a slowed decline in eGFR (97). The 2025 ADA guidelines recommend the use of SGLT2 inhibitors for patients with T2DM and DKD when the eGFR is ≥20 mL/min/1.73m². This recommendation is primarily based on the proven efficacy of SGLT2 inhibitors in reducing the progression of CKD and the risk of major adverse cardiovascular events.

SGLT2 inhibitors also modulate mitochondrial-associated endoplasmic reticulum membranes (MAMs), critical platforms for calcium signaling and lipid metabolism at ER-mitochondria contact sites. In diabetic podocytes, hyperglycemia-induced MAMs hyperplasia exacerbates mitochondrial dysfunction and ER stress, promoting podocyte apoptosis and DAMP release. Empagliflozin treatment and podocyte-specific SGLT2 knockout reduced MAMs formation via AMPK activation, thereby alleviating mitochondrial Ca²^+^ overload and NLRP3 inflammasome activation (98). This mechanism directly connects SGLT2 inhibition with the suppression of macrophage-recruiting cytokines and T cell infiltration, as MAMs-derived ROS and calcium flux are key drivers of immune cell activation in DKD. Emerging evidence suggests that the renoprotective effects of SGLT2 inhibitors extend beyond diabetic nephropathy to autoimmune kidney diseases. In the case of lupus nephritis (LN), treatment with empagliflozin has been shown to reduce glomerular and tubulointerstitial injury. Mechanistically, SGLT2 inhibitors enhance podocyte autophagy by suppressing mTORC1 activity and reduce NLRP3 inflammasome-mediated inflammation, thereby preserving synaptopodin expression and podocyte integrity. These findings underscore the pleiotropic effects of SGLT2 inhibitors in autoimmune settings, where they protect podocytes through dual mechanisms of autophagy promotion and inflammation attenuation (99).

#### 4.1.3 GLP-1 receptor agonists

Glucagon-like peptide-1 receptor agonists (GLP-1 RAs), traditionally recognized for glycemic regulation and weight management, exhibit renoprotective effects in DKD through multifactorial mechanisms. By enhancing glucose-dependent insulin secretion and suppressing glucagon release, these agents reduce chronic hyperglycemia-induced oxidative stress and AGE accumulation, thereby attenuating glomerular basement membrane thickening and albuminuria (100). Clinical trials, such as the Assessment of the Effects of Efpeglenatide on Cardiovascular and Renal Outcomes (AMPLITUDE-O) trial, have demonstrated that GLP-1 RAs reduce the incidence of macroalbuminuria in patients with DKD, independent of glycemic control (101). GLP-1 RAs exert their effects through various mechanisms, including the modulation of renal hemodynamics, reduction of inflammation, and inhibition of fibrosis within the kidneys. Furthermore, GLP-1 RAs provide significant cardiovascular benefits, which are mediated by a combination of pathways. They effectively lower blood pressure, improve lipid profiles by increasing high-density lipoprotein cholesterol (HDL-C) levels and decreasing triglycerides, and also promote weight loss. Additionally, GLP-1 RAs enhance vascular endothelial function, mitigate systemic inflammation, and positively impact cardiac function. Collectively, these effects contribute to the mitigation of systemic risk in diabetic nephropathy, thereby reducing the overall burden of cardiovascular disease, a major complication in patients with DKD.

Emerging evidence highlights their immunomodulatory role in the pathogenesis of DKD. GLP-1 RAs inhibit the TLR4/MYD88/NF-κB signaling pathway in renal tubular cells, suppressing NLRP3 inflammasome activation and subsequent IL-1β/IL-18 release, which are key drivers of interstitial inflammation (102). Preclinical studies indicate that liraglutide directly decreases renal macrophage infiltration by downregulating the expression of MCP-1 and CXCL1 chemokines, thereby limiting the recruitment of monocytes to injured glomeruli (103). Furthermore, GLP-1 RAs enhance Treg activity while inhibiting Th17 differentiation, rebalancing the Th17/Treg axis to mitigate podocyte injury and fibrosis (104). Semaglutide has been shown to decrease urinary TGF-β1 levels, indicating reduced fibrotic signaling in clinical cohorts (105). Guidelines from the American Diabetes Association (ADA) and the European Association for the Study of Diabetes (EASD) prioritize the use of GLP-1 RAs for patients with DKD who have cardiovascular comorbidities. This prioritization is primarily due to the well-established ability of GLP-1 RAs to reduce the risk of major adverse cardiovascular events, as evidenced by large-scale clinical trials. Emerging evidence also suggests that GLP-1 RAs may have beneficial effects on immune-mediated kidney injury, potentially by modulating inflammatory pathways within the kidney.

#### 4.1.4 Metformin

Metformin, a first-line therapy for T2DM, exerts renoprotective effects in DKD through its classical metabolic actions. By activating adenosine monophosphate-activated protein kinase (AMPK), metformin suppresses hepatic gluconeogenesis, reduces insulin resistance, and mitigates hyperglycemia-induced mitochondrial oxidative stress. These mechanisms collectively decrease AGE deposition and glomerular hyperfiltration, slowing albuminuria progression. The United Kingdom Prospective Diabetes Study (UKPDS) demonstrated that metformin reduced diabetes-related complications, including kidney disease, in overweight patients (106). While traditionally contraindicated in advanced chronic kidney disease (CKD), updated Kidney Disease: Improving Global Outcomes (KDIGO) 2024 guidelines now cautiously endorse metformin for DKD patients with eGFR ≥30 mL/min/1.73m² due to refined risk-benefit assessments.

Beyond its glucose-lowering effects, metformin modulates immune-inflammatory pathways in DKD. It inhibits the NLRP3 inflammasome in renal macrophages by enhancing AMPK-mediated autophagy, thereby reducing caspase-1 activation and the secretion of IL-1β/IL-18, which are key mediators of tubulointerstitial injury (107). Metformin also downregulates TLR4-NF-κB signaling in podocytes, decreasing pro-inflammatory cytokine production and MCP-1-driven macrophage infiltration (108). Preclinical studies indicate that metformin promotes M2 macrophage polarization through STAT6 activation, which facilitates tissue repair and attenuates TGF-β1-mediated renal fibrosis (109). In diabetic mouse models, metformin has been shown to reduce renal NLRP3 expression and urinary MCP-1 levels.

#### 4.1.5 DPP-4 inhibitors

Dipeptidyl Peptidase-4 (DPP-4) inhibitors, primarily used for glycemic control in T2DM, exert renoprotective effects through metabolic stabilization. By inhibiting the degradation of incretins, these agents enhance insulin secretion, suppress glucagon release, and reduce postprandial hyperglycemia-dependent oxidative stress, thereby attenuating AGE accumulation and glomerular endothelial dysfunction. Although traditional clinical trials, such as the Saxagliptin Assessment of Vascular Outcomes Recorded in Patients with Diabetes Mellitus-Thrombolysis in Myocardial Infarction (SAVOR-TIMI) 53 study, demonstrated neutral effects on the progression of DKD in non-selective cohorts, certain subgroup analyses indicated a potential reduction in albuminuria among early-stage DKD patients (110). Emerging evidence underscores the immunomodulatory properties of DPP-4 inhibitors in the pathogenesis of DKD. Preclinical studies indicate that DPP-4 inhibitors suppress NLRP3, TLR4, and IL-1β in human macrophages by inhibiting PKC activity (111). They also inhibit CD26-mediated cleavage of chemokines, thereby limiting the recruitment of monocytes/macrophages into the renal parenchyma and subsequent interstitial fibrosis. When considering the use of DPP-4 inhibitors in patients with DKD, factors such as the patient’s overall health, comorbidities, and potential drug-specific effects on renal function should be thoroughly evaluated. Although there is no explicit recommendation regarding their use based on residual β-cell function in DKD patients, individualized treatment decisions remain crucial, taking into account aspects such as glycemic control, cardiovascular risk, and tolerability.

#### 4.1.6 ACEI/ARB

Angiotensin-converting enzyme inhibitors (ACEIs) and angiotensin II receptor blockers (ARBs) remain cornerstone therapies for DKD by targeting the RAAS. These agents reduce glomerular hypertension through vasodilation of efferent arterioles, lowering intraglomerular pressure and albuminuria. Landmark trials, including the Reduction of Endpoints in NIDDM with the Angiotensin II Antagonist Losartan (RENAAL) and Irbesartan Diabetic Nephropathy Trial (IDNT) studies, demonstrated that ARBs reduce progression to end-stage kidney disease (ESKD) in type 2 diabetes mellitus (T2DM) patients with proteinuria (112). Guidelines from organizations such as the ADA recommend the use of ACEIs/ARBs in patients with diabetes mellitus and hypertension accompanied by albuminuria to delay the progression of kidney disease. The evidence is strongest in patients with severe albuminuria. In addition to hemodynamic effects, ACEIs/ARBs mitigate fibrotic pathways by suppressing angiotensin II-mediated TGF-β1 overexpression in podocytes and tubular cells. Recent evidence highlights their immunomodulatory action in DKD. ACEIs/ARBs, such as enalapril and losartan, differentially inhibit inflammatory responses by suppressing oxidative stress-induced NF-κB activation in the kidneys of aged rats (113). Clinical studies indicate that ramipril decreases urinary MCP-1 excretion in patients with DKD, which correlates with stabilized renal function (114). Guidelines emphasize its dual hemodynamic and anti-inflammatory effects, recommending early initiation even in normotensive DKD patients with microalbuminuria.

#### 4.1.7 MRAs

Selective mineralocorticoid receptor antagonists (MRAs) like finerenone show superior renoprotection in DKD by blocking aldosterone-induced MR overactivation. Unlike non-selective agents, finerenone exhibits higher tissue selectivity and lower hyperkalemia risk while effectively reducing albuminuria through hemodynamic and antifibrotic mechanisms (115). Phase III trials (FIDELIO-DKD, FIGARO-DKD) demonstrated finerenone reduced urinary albumin-to-creatinine ratio and delayed renal composite endpoints in T2D patients with CKD stages 3–4 (116). MRAs as add-on therapy for DKD patients with persistent albuminuria despite RAS blockade and SGLT2 inhibitors use, particularly advocating selective agents for their safer cardiorenal profile. Mechanistically, they attenuate aldosterone-driven sodium retention, glomerular hyperfiltration, and TGF-β1-mediated collagen deposition in mesangial cells (117).

Novel insights reveal that MRAs modulate immune-inflammatory pathways in DKD. Finerenone downregulates MR-NF-κB crosstalk in renal fibroblasts, decreasing MCP-1-dependent monocyte recruitment and interstitial macrophage infiltration. Additionally, selective MRAs restore podocyte autophagy via AMPK activation, mitigating immune-mediated cytoskeletal disruption and slit diaphragm damage (118). Non-selective MRAs, such as spironolactone, also reduce TLR4-mediated pro-inflammatory cytokine release; however, their lack of receptor specificity limits their clinical utility in advanced CKD. Finerenone offers dual benefits of antifibrotic and immunomodulatory effects, making it a recommended option for prioritized use in DKD patients with coexisting cardiovascular risks.

#### 4.1.8 Lipid-lowering agents (statins and PCSK9 inhibitors)

Statins, including atorvastatin and rosuvastatin, primarily exert renoprotective effects in DKD by lowering low-density lipoprotein (LDL) cholesterol through the inhibition of 3-Hydroxy-3-methylglutaryl coenzyme A (HMG-CoA) reductase. This action mitigates lipid-induced glomerulosclerosis and tubular epithelial apoptosis. *Post hoc* analyses of clinical trials, such as CARDS and PLANET I/II, have demonstrated that intensive statin therapy reduces albuminuria and decelerates the decline in eGFR in diabetic patients with moderate CKD (119, 120). Beyond their lipid-lowering effects, statins stabilize the podocyte actin cytoskeleton by enhancing nephrin expression and inhibit mesangial matrix expansion through the downregulation of TGF-β1.

Emerging evidence underscores the immunomodulatory effects of statins in DKD. By inhibiting the production of isoprenoids from the mevalonate pathway, statins prevent the activation of the ras homolog gene family, member A (RhoA)/rho-associated protein kinase (ROCK) in renal macrophages, thereby reducing the assembly of the NLRP3 inflammasome and subsequent production of IL-1β/IL-18 (121, 122). They suppress TLR4/NF-κB signaling in proximal tubular cells, which decreases the secretion of MCP-1 and TNF-α—critical mediators of monocyte infiltration. Clinical studies indicate that rosuvastatin reduces urinary IL-6 and CD40 ligand levels in patients with DKD, correlating with a reduction in macrophage density in renal biopsies (123). Proprotein convertase subtilisin/kexin type 9 (PCSK9) inhibitors further augment immunomodulation by promoting hepatic LDL receptor recycling, thereby diminishing oxidized LDL-induced NLRP3 activation in glomerular endothelial cells (124). However, excessive lowering of LDL may paradoxically worsen renal inflammation by impairing reverse cholesterol transport.

#### 4.1.9 Antioxidant therapies

Antioxidant therapies are a crucial strategy for preventing immune-mediated renal injury in DKD by targeting mitochondrial oxidative stress and suppressing cytokine storms. Preclinical and early clinical trials have shown that various antioxidants exert their effects through different mechanisms. Bardoxolone methyl (BAR), an Nrf2 agonist, mitigates inflammation by activating nuclear factor erythroid-derived 2-related factor 2 (Nrf2) and inhibiting NF-κB, thereby decreasing mitochondrial ROS production and preventing the NF-κB-mediated release of pro-inflammatory cytokines through the upregulation of antioxidant enzymes. A phase 3 bardoxolone methyl evaluation in patients with chronic kidney disease and T2DM (BEACON) trial (NCT01611569) in 2185 patients with T2DM and stage 4 chronic kidney disease showed that BAR transiently increased eGFR and reduced urine albumin-to-creatinine ratio. *Post hoc* analyses demonstrated that alterations in albuminuria were closely associated with eGFR dynamics and diminished tubulointerstitial inflammation. Specifically, bardoxolone significantly lowered albuminuria when adjusted for eGFR, thereby challenging the traditional belief that increased albuminuria universally signifies kidney injury (125).

Mitochondria-targeted antioxidant MitoQ, a mitochondria-specific coenzyme Q10 analog, alleviates podocyte apoptosis and tubular epithelial necrosis in diabetic kidney disease by scavenging mitochondrial ROS and restoring mitophagy via the Nrf2/PINK1 signaling pathway. Animal studies in db/db mice revealed that MitoQ reversed mitochondrial fragmentation, reduced ROS overproduction, and restored PINK1/Parkin-mediated mitophagy, accompanied by suppressed NLRP3 inflammasome activation and decreased secretion of pro-inflammatory cytokines. In high-glucose-treated HK-2 tubular cells, MitoQ upregulated Nrf2 translocation, inhibited Kelch-like ECH-associated protein 1 (Keap1), and blocked M1 macrophage polarization and Th17 cell infiltration through mitochondrial quality control mechanisms, establishing a link between mitochondrial redox balance and immune cell dysregulation in DKD (126, 127).

Natural antioxidant alpha-lipoic acid (ALA) and its derivative alpha lipoamide (ALM) exhibit renoprotective effects in DKD (128). In db/db diabetic mice and high-glucose-treated rat renal tubular epithelial cell line NRK-52E, ALA/ALM enhances mitochondrial function by diminishing reactive oxygen species and restoring mitofusin 1 (Mfn1)/dynamin-related protein 1 (Drp1)-mediated mitochondrial dynamics. Mechanistically, ALM activates retinoid X receptor-α (RXRα) through stable hydrogen bond formation, thereby upregulating caudal-type homeobox transcription factor 2 (CDX2) and cystic fibrosis transmembrane conductance regulator (CFTR), while concurrently suppressing β-catenin/Snail-mediated epithelial-mesenchymal transition (EMT). This dual action effectively inhibits extracellular matrix deposition and tubulointerstitial fibrosis (129). These therapies exert a triple effect of “antioxidation, anti-inflammation, and immunomodulation,” not only resolving mitochondrial dysfunction but also directly inhibiting excessive immune cell activation, offering novel directions for the precision treatment of DKD.

### 4.2 Renal replacement therapies in DKD: dialysis and kidney transplantation

Renal replacement therapies, which encompass dialysis and kidney transplantation, remain crucial for managing end-stage DKD. Both hemodialysis and peritoneal dialysis alleviate uremia by filtering toxins and maintaining electrolyte balance, yet they do not address residual immune-mediated injury. It is noteworthy that the biocompatibility of dialysis membranes affects systemic inflammation; cellulose-based membranes provoke complement activation and monocyte IL-6 release, thereby exacerbating DKD-related inflammaging (130). Kidney transplantation provides superior outcomes by restoring renal function; however, post-transplant immunosuppressants may paradoxically exacerbate diabetic microvascular damage through NF-κB-mediated endothelial dysfunction (131).

Immunologically, dialysis exacerbates innate immune dysregulation by promoting oxidative stress-driven NLRP3 activation in circulating monocytes, increasing IL-1β levels. Persistent TLR signaling in dialysis patients accelerates inflammasome priming, perpetuating renal fibrosis in remnant nephrons (132, 133). Transplantation modulates adaptive immunity by depleting alloreactive T-cells, but it may also impair macrophage polarization, hindering tissue repair. Emerging immunomodulatory strategies, such as cytokine adsorbent columns during dialysis or belatacept-based regimens in transplantation, aim to achieve a balance in immune homeostasis (134). Monitoring immune senescence markers to optimize replacement therapy timing.

### 4.3 Traditional Chinese Medicine in DKD management

Traditional Chinese Medicine (TCM) demonstrates renoprotective effects by modulating multiple targets, primarily through the regulation of oxidative stress and the mitigation of glomerulotubular fibrosis. Active compounds, such as astragaloside IV, attenuate diabetes-related activation of protein kinase B (Akt)/mammalian target of rapamycin (mTOR), NF-κB, and extracellular signal-regulated kinases 1 and 2 (Erk1/2) signaling pathways, thereby decreasing podocyte apoptosis and albuminuria in preclinical models (135). Clinical trials of TCM formulations demonstrate synergistic effects with RAS inhibitors, slowing eGFR decline in DKD patients. TCM should be cautiously recognized as an adjuvant therapy, with an emphasis on the standardization of bioactive components and rigorous assessment of drug interactions.

TCM exerts immunomodulatory actions by targeting innate immune pathways. Berberine has demonstrated its anti-inflammatory effects through the activation of AMPK-dependent autophagy in adipose tissue macrophages (ATMs), which leads to a reduction in urinary IL-1β levels in diabetic mice (136). Astragalus polysaccharides may modulate the immunity of the host organism by activating the TLR4-mediated, MyD88-dependent signaling pathway (137). Notably, compounds from TCM, such as triptolide, inhibit TLR4/NF-κB signaling in tubular epithelial cells, thereby reducing CCL2-mediated monocyte recruitment (138). Nevertheless, the risks of non-specific immunosuppression remain; excessive use of Tripterygium wilfordii is linked to CD4+ T-cell lymphopenia.

### 4.4 Immunotherapy for DKD

#### 4.4.1 Cell therapy

##### 4.4.1.1 Treg therapy

**Figure 4:**
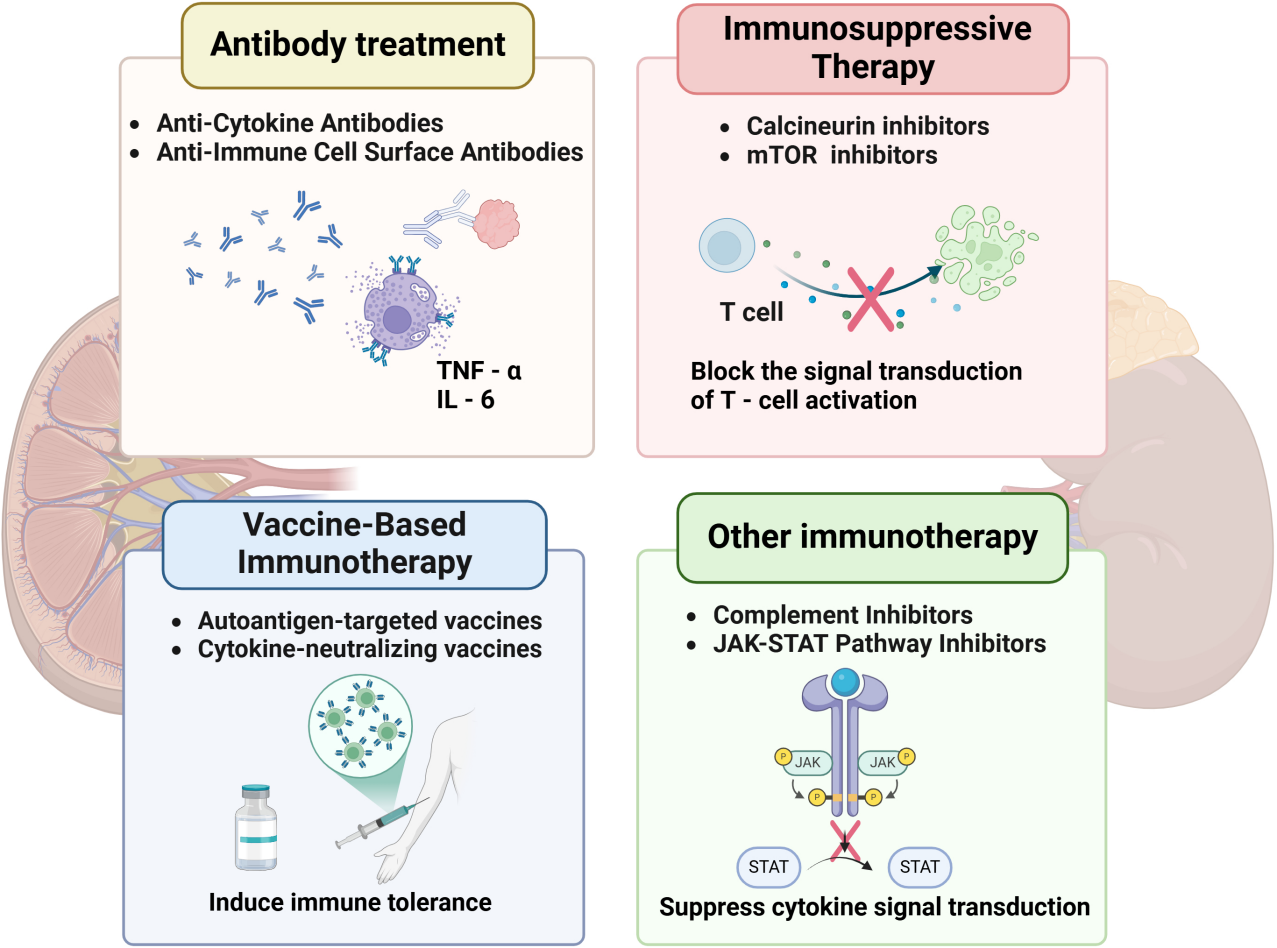
Immune-related therapies for DKD.

Tregs play a pivotal role in mitigating immune-mediated renal injury in DKD by suppressing effector T cell activation and restoring immune tolerance. In diabetic environments, chronic hyperglycemia disrupts Treg function by downregulating FoxP3 expression and impairing IL-35-mediated immunosuppression, exacerbating Th17-driven interstitial inflammation. Adoptive Treg transfer—expanding autologous Tregs ex vivo and reinfusing them into patients—has shown promise in preclinical studies (139). Human placenta-derived mesenchymal stem cells (PMSCs) have been shown to enhance renal function and alleviate pathological damage in rats with DKD, while also modulating the Th17/Treg balance via the PD-1/PD-L1 pathway (24). Mechanistically, Tregs inhibit Th1/Th17 polarization through cytotoxic T lymphocyte-associated protein 4 (CTLA-4) and TGF-β signaling, while promoting anti-inflammatory macrophage (M2) polarization via IL-10 secretion (140). In animal models of autoimmune diabetes, Treg therapy has demonstrated safety, with evidence indicating a reduction in systemic inflammation and the preservation of β-cell function (141). Further optimization of tissue-targeted Treg delivery could enhance therapeutic precision while minimizing off-target immunosuppression. (**Figure 4**).

##### 4.4.1.2 Mesenchymal stem cell therapy

Mesenchymal stem cells (MSCs) exert dual regenerative and immunomodulatory effects in DKD by differentiating into renal progenitor cells and secreting paracrine factors. In hyperglycemic conditions, MSCs suppress pro-inflammatory cytokines through NF-κB inhibition and enhance antioxidant defenses by activating the nuclear factor erythroid 2-related factor 2 (Nrf2) pathway. Notably, MSC-derived extracellular vesicles (EVs) transfer miR-let7c to tubular epithelial cells, mitigating mitochondrial ROS overproduction and apoptosis. Immunologically, MSCs inhibit dendritic cell maturation and B cell antibody production while promoting Treg expansion via the prostaglandin E2 (PGE2)/indoleamine 2,3-dioxygenase (IDO) axis. In diabetic mice, intrarenal MSC administration has been shown to reduce renal M1 macrophages and upregulate reparative M2 markers (56). Phase I trials have demonstrated the safety of MSCs in patients with DKD, with transient stabilization of eGFR and reduced urinary IL-18 levels (142). Challenges include inefficient homing and the potential for fibrotic transformation under chronic inflammation, necessitating the engineering of MSCs with enhanced renal tropism.

#### 4.4.2 Antibody treatment

##### 4.4.2.1 Anti-cytokine antibodies

Targeting pro-inflammatory cytokines such as TNF-α and IL-6 has emerged as a promising strategy for mitigating immune-mediated renal injury in DKD (143). TNF-α, a central mediator of the inflammatory cascade, exacerbates renal damage by promoting podocyte apoptosis, recruiting M1 macrophages, and activating the NF-κB pathway, which amplifies downstream cytokine production. Anti-TNF-α monoclonal antibodies neutralize soluble TNF-α, preventing its binding to TNFR1/2 receptors. In experimental DKD models, infliximab reduced albuminuria and attenuated glomerulosclerosis via suppression of ICAM-1-mediated leukocyte adhesion. Similarly, IL-6 drives renal fibrosis by activating STAT3 in fibroblasts and upregulating vascular cell adhesion molecule-1 (VCAM-1) expression in endothelial cells (144). Tocilizumab, an anti-IL-6 receptor antibody, inhibited IL-6 trans-signaling in diabetic mice, decreasing renal collagen deposition and improving tubular integrity (145). However, systemic immunosuppression risks limit clinical translation, necessitating kidney-targeted delivery systems.

##### 4.4.2.2 Anti-immune cell surface antibodies

B cell depletion therapy employing anti-CD20 antibodies presents a novel strategy for modulating adaptive immunity in DKD. CD20+ B cells play a role in renal injury through the production of autoantibodies and the release of pro-inflammatory cytokines. Rituximab selectively depletes CD20+ B cells while preserving Bregs, thereby restoring immune tolerance. Clinical trials have demonstrated that rituximab can reduce proteinuria in patients with nephropathy and concurrent autoimmune disorders (146), correlating with decreased renal IL-6 and macrophage infiltration. Preclinical studies in diabetic rats demonstrated that rituximab blunted complement activation and preserved podocyte density (147). Nonetheless, concerns remain about hypogammaglobulinemia and compromised pathogen defense in immunocompromised individuals with diabetes. Emerging bispecific antibodies that target CD20 and inhibitory checkpoints could enhance therapeutic precision by localizing immunosuppressive effects specifically to inflamed kidneys.

#### 4.4.3 Immunosuppressive therapy

##### 4.4.3.1 Calcineurin inhibitors

Calcineurin inhibitors (CNIs), such as cyclosporine A and tacrolimus, mitigate immune-mediated renal injury in DKD by suppressing T cell activation through the blockade of nuclear factor of activated T cells (NFAT) signaling. Mechanistically, CNIs inhibit calcineurin, preventing the dephosphorylation and nuclear translocation of NFAT—a critical step in cytokine transcription. Tacrolimus has been utilized in the management of immune-mediated and genetic-mediated nephropathies, with an emphasis on the restoration of podocyte cytoskeletal integrity and inhibition of apoptosis. In preclinical studies, researchers constructed Arginine-Glycine-Aspartic acid-Human Serum Albumin-Tacrolimus (RGD-HSA-TAC) nanoparticles to target the delivery of tacrolimus to podocytes, which resulted in a reduction of podocyte injury and albuminuria in mice with diabetic kidney disease (148). However, dose-dependent nephrotoxicity and hypertension limit long-term use, necessitating strict therapeutic drug monitoring. Emerging modified-release formulations aim to improve renal distribution and minimize systemic side effects.

##### 4.4.3.2 mTOR inhibitors

mTOR inhibitors, such as sirolimus, mitigate renal inflammation and fibrosis through the dual inhibition of adaptive and innate immunity. The mTOR complexes (mTORC1/2) control the proliferation of T/B cells and the polarization of macrophages; inhibition leads to a shift in macrophages from a pro-inflammatory M1 phenotype to a reparative M2 phenotype, mediated by STAT6 activation. Preclinical models demonstrate that sirolimus reduces renal collagen IV deposition and suppresses VEGF-induced glomerular hypertrophy in diabetic rats (149). Paradoxically, mTOR inhibitors may exacerbate podocyte apoptosis in advanced DKD due to disrupted autophagy, highlighting the need for patient stratification based on disease stage.

#### 4.4.4 Vaccine-based immunotherapy

##### 4.4.4.1 Autoantigen-targeted vaccines

Autoantigen-targeted vaccines represent a novel therapeutic approach for DKD by restoring immune tolerance to renal-specific antigens. In cases of diabetic nephropathy, chronic hyperglycemia induces the formation of autoantibodies against podocyte antigens, which cross-react with glomerular components, triggering complement activation and foot process effacement. Tolerogenic vaccines, loaded with podocyte-derived peptides, prime dendritic cells to induce antigen-specific Tregs rather than effector T cells (150). Preclinical studies have shown that these vaccines enhance renal infiltration of FoxP3+ Tregs and decrease anti-podocyte IgG titers in streptozotocin-induced diabetic mice (151). Modified liposome vectors conjugated with CTLA-4-Ig fusion proteins enhance vaccine efficacy by blocking CD28/B7 costimulatory signals, thereby inhibiting Th17 differentiation and promoting the production of IL-10-producing Tregs. Multi-epitope vaccines, which incorporate B cell epitopes from various podocyte proteins, are under development to broaden immune tolerance.

##### 4.4.4.2 Cytokine-neutralizing vaccines

Cytokine-neutralizing vaccines provide a complementary strategy by targeting humoral immunity against pathogenic inflammatory mediators. Vaccines conjugated with TNF-α elicit neutralizing antibodies that lower circulating TNF-α levels in primate models, achieving effects comparable to those of monoclonal antibody therapies, but with the added benefit of sustained effects lasting at least 12 weeks post-immunization. This method circumvents the need for frequent dosing, which is often necessary for biologic agents such as infliximab. Additionally, the macrophage-stimulating potential of Hsp70 family proteins, when combined with the proinsulin B-chain peptide B11-23, may contribute to the immunodominance of this peptide in the development of beta cell-directed autoimmunity in type 1 diabetes (152). Challenges include cytokine redundancy within inflammatory networks and the potential for interference with physiological immune surveillance. Nanoparticle-delivered mRNA vaccines encoding soluble decoy receptors have shown promise in preclinical models by providing controlled cytokine neutralization without depleting essential immune effectors.

#### 4.4.5 Other immunotherapy

##### 4.4.5.1 Complement inhibitors

Complement activation is crucial in the progression of DKD, as it leads to the deposition of the membrane attack complex (MAC) and inflammation mediated by C5a. Eculizumab, a monoclonal antibody that targets complement protein C5, blocks the formation of MAC and decreases the release of C5a-driven NETs, thereby alleviating glomerular endothelial injury. Preclinical studies have shown that mice deficient in C3aR/C5aR exhibit significantly reduced proteinuria, lower renal IgA and C3 deposition, diminished histological damage, and reduced mesangial proliferation in a Sendai virus-induced IgA nephropathy (IgAN) model (153). MCC950, an inhibitor of the NLRP3 inflammasome, suppresses IL-1β production and maintains glomerular integrity through downstream complement regulatory effects. However, the increased risk of infection necessitates patient stratification based on biomarkers of complement activation. Novel nanocarriers delivering fusion proteins of complement receptor 2 and complement factor H (CR2-fH) facilitate renal-selective complement inhibition, thereby reducing the risks associated with systemic immunosuppression.

##### 4.4.5.2 JAK-STAT pathway inhibitors

Aberrant janus kinase-signal transducer and activator of transcription (JAK-STAT) signaling exacerbates renal inflammation in DKD by maintaining prolonged cytokine receptor activation. Baricitinib, a JAK1/2 inhibitor, diminishes STAT3 phosphorylation in diabetic kidney fibroblasts, thereby reducing collagen IV synthesis and tubular epithelial-mesenchymal transition. In db/db mouse models, Wogonin mitigates renal inflammation and fibrosis by upregulating suppressor of cytokine signaling 3 (SOCS3), which inhibits TLR4 and the JAK/STAT pathway (154). Selective tyrosine kinase 2 (TYK2) inhibitors show promise in preserving antiviral immunity while suppressing IFN-α/β-driven fibrosis (155). Thrombotic risks associated with JAK inhibitors require careful monitoring in diabetic populations with preexisting cardiovascular comorbidities.

##### 4.4.5.3 WNT signaling inhibitors

Wingless-type MMTV integration site family member (WNT) signaling inhibitors hold promise for regulating autophagy and immune cell polarization. β-catenin, a central mediator of the WNT pathway, is abnormally activated in DKD, promoting renal fibroblast proliferation and extracellular matrix deposition while inhibiting macrophage autophagy through the mTOR pathway, thereby inducing M1 polarization (156). The WNT inhibitor XAV939 enhances the expression of autophagy-related genes ATG5 and ATG7 in tubular epithelial cells by inhibiting β-catenin nuclear translocation, thereby reducing TNF-α-induced M1 macrophage recruitment (157, 158). Preclinical studies demonstrate that inhibition of WNT decreases renal WNT3a levels, improves autophagic flux, and attenuates glomerulosclerosis and interstitial fibrosis, which are closely correlated with reduced CD68+ macrophage infiltration and IL-17 secretion (159). Notably, SGLT2 inhibitors enhance autophagy by improving mitochondrial function, and their combined use with WNT inhibitors synergistically suppresses inflammatory cascades through autophagy-dependent mitochondrial quality control, establishing a dual protective mechanism of “metabolic regulation-immune remodeling” (160, 161).

## 5 Challenges in immunotherapy for DKD

The advancement of immune-targeted therapies for DKD faces multiple intertwined challenges. First, the complex pathogenesis, which involves crosstalk between hyperglycemia-driven metabolic disturbances, inflammatory cascades, and immune cell dysregulation, creates difficulty in identifying singular therapeutic targets. Second, broad-spectrum immunosuppressants, such as anti-TNF-α biologics, reduce albuminuria but inadvertently increase infection risks due to systemic immune suppression, which is exacerbated by the baseline immune dysfunction in diabetic patients. Thirdly, patient heterogeneity in terms of genetic predisposition, renal immune cell infiltration patterns, and gut microbiota composition results in varying therapeutic responses. Moreover, traditional biomarkers such as urinary albumin do not adequately capture dynamic immune changes, including macrophage polarization or complement activation (C5b-9), which can lead to delayed treatment adjustments. Finally, the design of long-term trials with clinically meaningful endpoints encounters logistical challenges, as demonstrated by the premature termination of numerous phase III studies due to insufficient enrollment or delayed therapeutic effects. To address these challenges, there is a need for kidney-specific drug delivery systems, multi-omics-guided patient stratification, and validated composite biomarkers that reflect both metabolic and immune remodeling.

## 6 Future directions for immune therapy

Advancing immune-targeted therapies for DKD necessitates the integration of precision medicine with technological innovation. Multi-omics approaches, including single-cell RNA sequencing and spatial proteomics, facilitate the stratification of patients based on dominant immune pathways, thereby guiding personalized therapies. Next-generation agents with renal-specific targeting are emerging: bispecific antibodies reduce extrarenal toxicity in preclinical models by selectively modulating macrophages, while gene-edited chimeric antigen receptor regulatory T cells (CAR-Tregs) restore immune tolerance by homing to injured glomeruli via integrin α8β1 ligands. Combinatorial strategies synergize immunomodulation with metabolic control—renal-tropic nanoparticles co-delivering SGLT2 inhibitors and NF-κB decoy oligos enhance glycocalyx repair and reduce IL-6 levels in diabetic primates. Real-time monitoring innovations, such as urinary exosomal miRNA panels and renal positron emission tomography-magnetic resonance imaging (PET-MRI) with C-X-C chemokine receptor 4 (CXCR4)-specific tracers, correlate with histologic inflammation and predict treatment response. Cross-disciplinary collaboration is critical; integrating immune checkpoint biology with tubulometabolic profiling identifies novel targets for antibody-drug conjugate (ADC) development. Decentralized clinical trial platforms, leveraging artificial intelligence (AI)-based kidney organoids, will accelerate the validation of these strategies.

## 7 Conclusions

Current therapies for DKD, which include glycemic control and RAS inhibition, are limited by their inability to completely halt immune-mediated renal inflammation and fibrosis. Non-specific anti-inflammatory approaches risk increasing infection susceptibility in immunocompromised diabetic populations, highlighting the inadequacy of broad immunosuppression. In contrast, emerging immunomodulatory strategies offer precision by disrupting pathogenic immune cascades while preserving host defense mechanisms. Preclinical studies demonstrate that therapies reducing renal macrophage infiltration or repolarizing dendritic cells alleviate albuminuria and glomerulosclerosis without systemic toxicity. Clinical trials of inflammasome inhibitors further validate the therapeutic potential of innate immune pathway modulation. Future research must prioritize biomarker-driven patient stratification and combinatorial regimens that integrate immune-specific agents with conventional therapies. By addressing the inflammatory axis central to DKD progression, these advances herald a paradigm shift toward precision medicine in renoprotection.

## Glossary

DKD: Diabetic kidney disease
RAAS: Renin-angiotensin-aldosterone system
TGF-β: Transforming growth factor beta
TNF-α: Tumor necrosis factor-alpha
STAT3: Signal transducer and activator of transcription 3
CCL20: Chemokine ligand 20
CCR6: Chemokine receptor 6
IFN-γ: Interferon gamma
Tregs: Regulatory T-cells
SGLT-2: Sodium-glucose cotransporter 2
BAR: Bardoxolone methyl
Nrf2: Nuclear factor erythroid-derived 2-related factor 2
BEACON: Bardoxolone methyl evaluation in patients with chronic kidney disease and type 2 diabetes
ALA: Alpha-lipoic acid
ALM: Alpha lipoamide
NRK-52E: Rat renal tubular epithelial cell line NRK-52E
Mfn1: Mitofusin 1
Drp1: Dynamin-related protein 1
RXRα: Retinoid X receptor-α
CDX2: Caudal-type homeobox transcription factor 2
CFTR: Cystic fibrosis transmembrane conductance regulator
EMT: Epithelial-mesenchymal transition
DAG: Diacylglycerol
PAMPs: Pathogen-associated molecular patterns
ULK1: Unc-51-like kinase 1
DAMPs: Damage-associated molecular patterns
WNT: Wingless-type MMTV integration site family member
HMGB1: High-mobility group box 1
MAMs: Mitochondrial-associated endoplasmic reticulum membranes
LN: Lupus nephritis
mTOR: Mammalian target of rapamycin
NLRP3: NOD-like receptor protein 3
IL-1β: Interleukin-1β
CCR2: Chemokine receptor 2
MCP-1: Monocyte chemoattractant protein 1
AGEs: Advanced glycation end products
NF-κB: Nuclear factor-κB
ROS: Reactive oxygen species
GBM: Glomerular basement membrane
FFAs: Free fatty acids
PPARs: Peroxisome proliferator-activated receptors
SREBPs: Sterol regulatory element-binding proteins
NOX: NADPH oxidase
MDA: Malondialdehyde
4-HNE: 4-hydroxynonenal
PKC: Hyperactivation of protein kinase C
VEGF: Vascular endothelial growth factor
8-OHdG: 8-hydroxy-2’-deoxyguanosine
ADA: American Diabetes Association
HIF-1α: Hypoxia-inducible factor-1α
CTLs: Cytotoxic T cells
NADPH: Nicotinamide adenine dinucleotide phosphate
T2DM: Type 2 diabetes mellitus
DCs: Dendritic cells
CDCs: Conventional DCs
PDCs: Plasmacytoid DCs
APCs: Antigen-presenting cells
MHC-II: Major Histocompatibility Complex Class II
Bregs: regulatory B cells
PD-L1: Programmed death-ligand 1
FcγRIIb: Fc Gamma Receptor IIb
ICAM-1: lntercellular adhesion molecule-1
C3a: Complement component 3a
DCCT: Diabetes Control and Complications Trial
RAGE: Receptor for advanced glycation end products
PI3K/AKT: Phosphatidylinositol 3-kinase/protein kinase B
CREDENCE: Canagliflozin and Renal Endpoints in Diabetes with Established Nephropathy Clinical Evaluation
NETs: Neutrophil extracellular traps
eGFR: Estimated glomerular filtration rate
GLP-1: RAs Glucagon-like peptide-1 receptor agonists
AMPLITUDE-O: Assessment of the Effects of Efpeglenatide on Cardiovascular and Renal Outcomes
HDL-C: High-density lipoprotein cholesterol
EASD: European Association for the Study of Diabetes
AMPK: Adenosine monophosphate-activated protein kinase
UKPDS: United Kingdom Prospective Diabetes Study
CKD: Chronic kidney disease
KDIGO: Kidney Disease: Improving Global Outcomes
TLR4: Toll-like receptor 4
DPP-4: Dipeptidyl Peptidase-4
SAVOR-TIMI: Saxagliptin Assessment of Vascular Outcomes Recorded in Patients with Diabetes Mellitus-Thrombolysis in Myocardial Infarction
ACEIs: Angiotensin-converting enzyme inhibitors
ARBs: Angiotensin II receptor blockers
RENAAL: Angiotensin II Antagonist Losartan
IDNT: Irbesartan Diabetic Nephropathy Trial
ESKD: End-stage kidney disease
MRAs: Mineralocorticoid receptor antagonists
LDL: Low-density lipoprotein
HMG-CoA: 3-Hydroxy-3-methylglutaryl coenzyme
RhoA: Ras homolog gene family, member A
ROCK: Rho-associated protein kinase
PCSK9: Proprotein convertase subtilisin/kexin type 9
TCM: Traditional Chinese Medicine
Erk1/2: Extracellular signal-regulated kinases 1 and 2
ATMs: Adipose tissue macrophages
PMSCs: Placenta-derived mesenchymal stem cells
CTLA-4: Cytotoxic T lymphocyte-associated protein 4
MSCs: Mesenchymal stem cells
Akt: Protein kinase B
mTOR: Mammalian target of rapamycin
Nrf2: Nuclear factor erythroid 2-related factor 2
EVs: Extracellular vesicles
PGE2: Prostaglandin E2
IDO: Indoleamine 2,3-dioxygenase
VCAM-1: Vascular cell adhesion molecule-1
CNIs: Calcineurin inhibitors
RGD-HSA-TAC: Arginine-Glycine-Aspartic acid-Human Serum Albumin-Tacrolimus
NFAT: Nuclear factor of activated T cells
MAC: Membrane attack complex
IgAN: IgA nephropathy
CR2-fH: Complement receptor 2 and complement factor H
JAK-STAT: Janus kinase-signal transducer and activator of transcription
SOCS3: Suppressor of cytokine signaling 3
TYK2: Tyrosine kinase 2
CAR-Tregs: Chimeric antigen receptor regulatory T cells
PET-MRI: Positron emission tomography-magnetic resonance imaging
CXCR4: C-X-C chemokine receptor 4
ADC: Antibody-drug conjugate
AI: Artificial intelligence.
